# Temporal Convolutional Neural Network Analysis of Magnetocardiography Signals for Detection of Pulmonary Hypertension

**DOI:** 10.3390/bioengineering13070736

**Published:** 2026-06-25

**Authors:** Yuankun Qi, Kai Ma, Xiaole Han, Dong Xu, Xu Zhang, Min Xiang

**Affiliations:** 1 Key Laboratory of Ultra-Weak Magnetic Field Measurement Technology, Ministry of Education, School of Instrumentation and Optoelectronic Engineering, Beihang University, Beijing 100191, China; yuankunqi@buaa.edu.cn (Y.Q.); mi8790@buaa.edu.cn (K.M.); zhang_xu@buaa.edu.cn (X.Z.); 2 Zhejiang Provincial Key Laboratory of Ultra-Weak Magnetic-Field Space and Applied Technology, Hangzhou 310051, China; hanxiaole@buaa.edu.cn (X.H.); mrwoodeast@126.com (D.X.); 3 Zhejiang Key Laboratory of Zero Magnetic Medicine, Hangzhou 310006, China; 4 State Key Laboratory of Traditional Chinese Medicine Syndrome, National Institute of Extremely-Weak Magnetic Field Infrastructure, Hangzhou 310028, China; 5 Hefei National Laboratory, Hefei 230088, China

**Keywords:** magnetocardiography, pulmonary hypertension, biomedical signal processing, machine learning, deep learning

## Abstract

Non-invasive methods used for PH detection in clinical practice have several limitations. The combination of high spatiotemporal sensitivity magnetocardiography (MCG) and artificial intelligence algorithms may offer an accurate approach for PH detection. In this study, we develop a convolutional neural network (CNN) model based on the 64-channel MCG time-series data. This exploratory study enrolled patients undergoing 64-channel MCG, including right-heart-catheterization confirmed PH patients and symptomatic controls with low echocardiographic probability of PH. After data preprocessing, a temporal CNN integrating MCG signals with age, sex, and body mass index was developed and compared with conventional machine learning models. The CNN model achieved strong discrimination, with area under the curve (AUC) values of 0.939 (95% confidence interval [CI]: 0.913–0.961) in the development out-of-fold evaluation and 0.974 (95% CI: 0.944–0.994) in the hold-out test set, outperforming conventional machine learning models. Decision curve analysis showed the greatest net benefit at clinically relevant thresholds. Attribution analysis indicated that spatial QRS morphology redistribution contributed substantially to PH classification. The temporal CNN model based on raw 64-channel MCG signals showed promising performance for non-invasive PH detection and outperformed conventional machine learning approaches in this exploratory single-center cohort enriched for PAH and CTEPH.

## 1. Introduction

Pulmonary hypertension (PH) is a progressive and often fatal condition affecting roughly 1% of the global population [1]. While invasive right heart catheterization (RHC) remains the diagnostic gold standard, its complication risks and steep technical demands restrict its use to specialized centers, rendering it impractical for routine screening [2,3]. Since the WHO formally defined PH in the 1970s, achieving precise, non-invasive assessment has remained a primary focus of clinical research. Transthoracic echocardiography (TTE) is currently the frontline non-invasive modality, used to evaluate right heart function and estimate pulmonary artery pressure via tricuspid regurgitation velocity (TRV) [2,4]. Yet, the technique demands a steep operator learning curve. Its reliability may be compromised in patients with poor acoustic windows or faint regurgitant jets, often rendering it insufficient for accurate clinical evaluation [5]. Standard electrocardiography (ECG) lacks the accuracy to detect early PH-induced electrical alterations, limiting its use as a standalone screening tool [6]. Although cardiac magnetic resonance (CMR) has attracted increasing attention in recent years, its widespread clinical adoption is constrained by prohibitive costs and prolonged acquisition times [7]. Consequently, there remains a clinical need for a novel, efficient, and non-invasive methodology for PH detection and assessment.

Recent advances in quantum sensing technology, magnetocardiography (MCG) based on spin-exchange relaxation-free optically pumped magnetometers (SERF-OPM) has provided a non-invasive method for evaluating cardiac electrophysiology [8]. Early MCG studies have confirmed that right ventricular overload can cause detectable electrophysiological alterations. These changes primarily manifest as aberrant morphologies and elevated intensities within pseudo-current density (PCD) maps [9]. Recently, researchers at Beihang University have developed machine learning models based on the spatial features of PCD and magnetic field (MF) maps, which outperformed traditional ECG in detecting PH [10,11]. However, these models are highly dependent on two-dimensional (2D) image reconstruction. While such visual images can effectively present the spatial distribution of the cardiac magnetic field, the generation process involves steps such as spatial interpolation, image transformation, and feature parameterization, which weaken the continuous temporal dynamic characteristics of the original signal. Therefore, analyzing only 2D images may not fully exploit the diagnostic potential of MCG. To address this issue, it is necessary to directly evaluate the original time-series signal before image construction.

Deep learning, particularly convolutional neural networks (CNNs), has substantially advanced the analysis of complex biomedical data by enabling data-driven feature learning [12]. In the field of PH, CNN-based models have been applied to ECG, chest X-rays, and CMR, where they have shown the capacity to identify subtle changes in cardiac electrophysiology, cardiopulmonary structure and right heart remodeling [13,14,15]. Given the high sensitivity of MCG to cardiac electrophysiological activity, modeling raw MCG signals using a temporal CNN may provide a complementary solution for capturing PH-related abnormalities and enhancing non-invasive detection capabilities. In this study, we aimed to develop a temporal CNN model for analyzing the raw signals of MCG, and to evaluate the performance differences between this model and machine learning algorithms.

## 2. Materials and Methods

### 2.1. Study Design and Population

This exploratory study enrolled patients who received MCG examination at Qilu Hospital of Shandong University between May 2024 and September 2025. The dedicated pulmonary vascular center at this hospital primarily focuses on pulmonary arterial hypertension (PAH, Group 1) and chronic thromboembolic pulmonary hypertension (CTEPH, Group 4). Therefore, this was a single-center PH cohort enriched for PAH and CTEPH, which did not represent the real-world PH etiological spectrum. Given the rare nature of these two classifications of PH, a two-pathway enrollment strategy for the PH cohort was implemented. The first pathway included inpatients who were admitted for RHC because of a high clinical suspicion of PH and a high probability of PH on TTE. The second pathway included established outpatients with a prior RHC-confirmed diagnosis of PH who attended routine follow-up. The control group consisted of patients with symptoms similar to those of patients with PH but in whom PH was considered unlikely based on a low echocardiographic probability of PH on TTE during the same period before MCG examination. Patients with an intermediate probability of PH on TTE, but without risk factors or associated conditions for PAH or CTEPH, were excluded because guideline recommendations support continued TTE follow-up rather than further assessment with RHC. Patients were excluded if they met any of the following criteria: (1) pregnancy; (2) implanted cardiac pacemakers; (3) surgical hardware in the chest; (4) claustrophobia.

RHC was performed using a 131F7 Swan-Ganz catheter (Edwards Life Sciences, Irvine, CA, USA). According to the 2022 ESC/ERS guidelines, PH was defined as an mPAP > 20 mmHg [2]. Suspected PH patients with an mPAP ≤ 20 mmHg were categorized into the control group. TTE examinations were performed using the GE Vivid E95 echocardiography machine (GE, Vingmed Ultrasound, Horten, Norway). In order to ensure data consistency, all RHC and TTE examinations were performed by the same team. The TTE probability of PH was assessed based on TRV and additional echocardiographic signs suggestive of PH. A low echocardiographic probability of PH was defined as a peak TRV ≤ 2.8 m/s, or an unmeasurable TRV, in the absence of additional echocardiographic signs suggestive of PH. A high echocardiographic probability of PH was defined as a peak TRV > 3.4 m/s, or a peak TRV of 2.9–3.4 m/s in the presence of additional echocardiographic signs [2].

### 2.2. MCG Data Acquisition and Preprocessing

**Figure 1:**
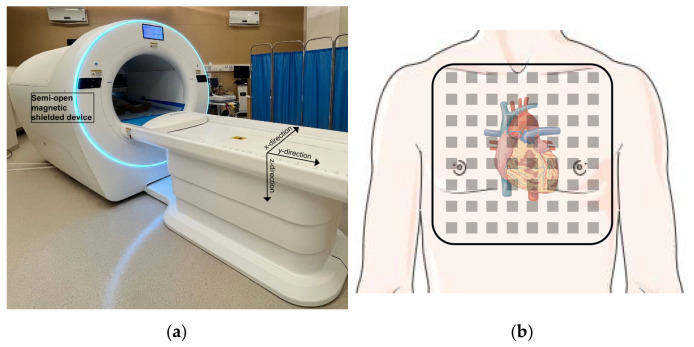
Schematic diagram of the MCG system: (**a**) the photograph of SERF-OPM-based MCG system; (**b**) the relative position of the sensor panel and the subject.

The MCG system with 64 SERF-OPM sensors was manufactured by Hangzhou National Institute of Extremely Weak Magnetic Field Infrastructure (Hangzhou, Zhejiang, China) (Figure 1a). The sensitivity of these sensors is below 15 fT/Hz^1/2^, and the sampling rate was 1000 Hz. The residual magnetic field inside the semi-open magnetic shielded device does not exceed 10 nT. After removing electronic devices and metal-containing clothing or accessories, each patient lay in a supine position within a semi-open magnetically shielded device for a 90 s MCG recording. The relative position of the sensor panel and the subject is shown in Figure 1b.

In order to reduce the artifacts and interference, the raw 64-channel MCG recordings were first preprocessed, including independent component analysis, empirical mode decomposition, and power-line notch filtering [16]. The preprocessed MCG data were organized as 64-channel signals with a sampling frequency of 1000 Hz. For CNN analysis, a standardized 1 s representative cardiac cycle template was generated for each recording. This 1 s MCG input was not directly cropped from an arbitrary time point of the 90 s recording. Instead, a representative cardiac cycle template was generated for each subject from the full 90 s MCG recording. In brief, after preprocessing, R-peaks were detected from the MCG signal, and the continuous recording was segmented into multiple cardiac cycles. The segmented beats were then assessed using morphology-based similarity analysis. Several beat templates were constructed, and the group of beats with the highest morphological consistency and the largest number of valid beats was selected to represent the dominant MCG activity during the recording. Beats with poor morphology, obvious noise contamination, unstable baseline, or atypical patterns were not included in the final template. The selected beats were aligned and averaged to generate a standardized 1 s representative cardiac cycle template. This procedure produced a fixed input dimension of 64 × 1000, corresponding to 64 MCG channels and 1000 temporal samples at a sampling frequency of 1000 Hz.

The generation of PCD and MF maps from preprocessed MCG recordings, and the extraction of feature parameters, were performed as described in our previous work [10,11].

### 2.3. Modeling

Due to the lack of an independent external validation cohort, we used a stratified strategy to assess the model’s internal generalizability. The full cohort was randomly split into a development set and a hold-out test set at a ratio of 8:2, with stratification according to the class label. The hold-out test set was not used for model training, hyperparameter tuning or threshold selection, and was reserved exclusively for final performance evaluation. In the development set, five-fold stratified cross-validation was performed to generate out-of-fold predicted probabilities. For each fold, four-fifths of the development samples were used for internal training, and the remaining one-fifth was used for internal validation. The optimal classification threshold was determined in the development set based on the maximum Youden index, using the out-of-fold predicted probabilities generated by five-fold cross-validation. This threshold was then fixed and applied to the hold-out test set.

**Figure 2:**
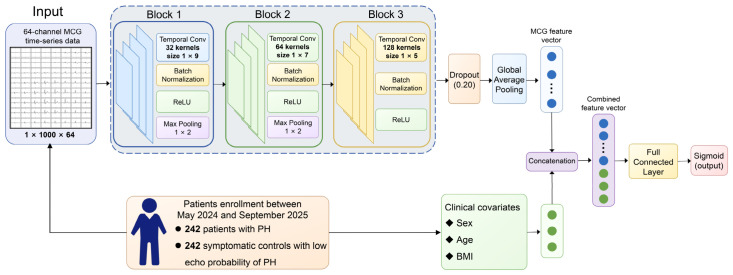
Workflow of the CNN model.

A binary classification model consisting of two input branches was developed: an MCG time-series convolutional branch and a clinical covariate (sex, age, and BMI) branch. In the MCG branch, 64-channel time-series data were used as input, with an input size of 1 × 1000 × 64. This branch comprised three temporal convolutional blocks. The detailed modelling workflow is shown in Figure 2. A dropout layer with a dropout rate of 0.20 was applied, and the MCG temporal feature representation was obtained using global average pooling. The three clinical covariates were concatenated with the MCG feature vector after global average pooling. The MCG representation derived from global average pooling was concatenated with the covariate tensor along the channel axis and passed to a single-neuron fully connected layer to obtain the binary classification logit. A sigmoid transformation was then used to convert this logit into the predicted probability. Model interpretation was performed using Integrated Gradients on the hold-out test set. Detailed information on model training and interpretation is provided in the Supplementary Methods Section.

To examine whether the performance of the proposed temporal CNN was dependent on a specific convolutional architecture, we performed supplementary sensitivity analyses using two additional lightweight deep learning models: the residual 1D-CNN and the multiscale 1D-CNN. These analyses were not intended to exhaustively benchmark all possible deep learning architectures, but to assess the robustness of direct raw MCG time-series modeling across different CNN-based structures. The same development and hold-out split, internal five-fold cross-validation strategy, threshold-selection procedure, and performance metrics were used for these supplementary models. Detailed information on the two additional deep learning models is provided in the Supplementary Methods.

According to the same stratified strategy, we evaluated the MCG feature parameters and same clinical covariates combined with several machine learning algorithms. The machine learning algorithms included support vector machine (SVM), k-nearest neighbor (KNN), random forest (RF) and logistic regression (LR). Furthermore, by employing soft voting techniques, SVM and RF were integrated to construct an ensemble model.

To evaluate the potential confounding caused by different input representations, supplementary ablation analyses were performed to evaluate the relative contributions of raw MCG time-series signals, clinical covariates, and feature representation strategies. The methods of the ablation analyses were presented in the Supplementary Materials Methods Section.

Finally, we used bootstrap resampling to estimate 95% confidence intervals (CIs) for model performance metrics based on fixed predicted probabilities. Bootstrap analysis was conducted separately for the out-of-fold predictions in the development set and the hold-out test predictions. The model was not retrained during each bootstrap iteration. Therefore, the 95% CIs in the results reflect the uncertainty of performance metrics conditional on the trained model. However, these intervals do not capture the full uncertainty related to model training, hyperparameter selection, or data splitting.

### 2.4. Statistical Analysis

Continuous variables with a normal distribution were summarized as mean ± standard deviation (M ± SD) and compared between groups using the independent-samples *t*-test. For continuous variables not following a normal distribution, they were described using the median [interquartile range], and inter-group comparisons were performed using the Mann–Whitney U test. Categorical variables were presented as counts and percentages (*n*, %) and compared using Pearson’s chi-square test when all expected frequencies were ≥5. If any expected frequency was <5, Fisher’s exact test was employed.

In all analyses, a two-sided *p* value < 0.05 was considered statistically significant. Receiver operating characteristic (ROC) analysis and decision curve analysis (DCA) were conducted. Model performance parameters were calculated based on the confusion matrix. These parameters included sensitivity, specificity, precision, accuracy, F1 score, and the areas under the receiver operating characteristic curves (AUCs). The DeLong test was used to compare AUCs between models. The test statistic was calculated as follows:

(1) $$Z=\frac{AUC_{1}\;-\;AUC_{2}}{\sqrt{Var\;(AUC_{1}\;-\;AUC_{2})\;}},$$

where $AUC_{1}$ and $AUC_{2}$ represent the AUCs of two compared models, and $Var(AUC_{1}-AUC_{2})$ was estimated using DeLong’s covariance approach.

All statistical analyses and model development were performed using MATLAB R2023b (MathWorks, Natick, MA, USA).

## 3. Results

### 3.1. Characteristics of Subjects

**Figure 3:**
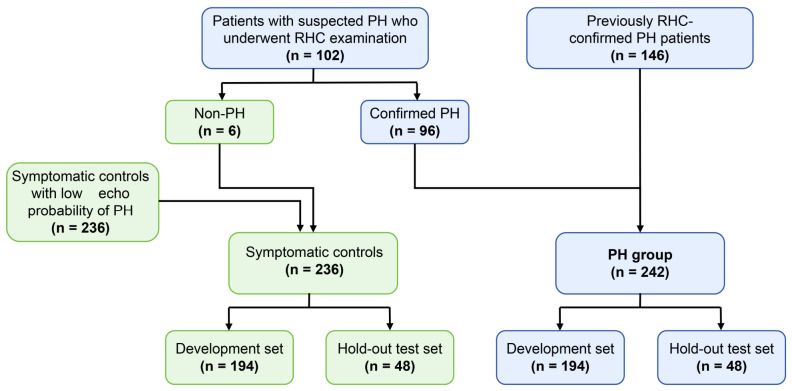
The patient flow diagram and data split.

A total of 248 patients with previously confirmed or suspected PH underwent MCG examination. After RHC assessment, six patients had an mPAP ≤ 20 mmHg and were not classified into the PH group; these patients were included in the symptomatic controls with low echocardiographic probability of PH (Figure 3). Consequently, a total of 242 patients with PH and 242 symptomatic controls were included in the final analysis. Among the PH patients, the majority were female (78.1%), and idiopathic pulmonary arterial hypertension was the predominant etiology (30.6%). Sex, age, and BMI differed significantly between PH patients and controls (Table S1). Additionally, most patients with PH exhibited relatively mild symptoms, categorized as WHO functional class I and II (71.5%). The detailed characteristics of PH patients are presented in Table 1. TTE parameters differed significantly between PH patients and controls (Table 2). Compared with controls, PH patients had significantly smaller left ventricular diameter. In contrast, parameters reflecting right heart enlargement were increased in PH patients, including right atrial area, right ventricular diameter, and main pulmonary artery diameter. These findings demonstrate significant right heart remodeling.

### 3.2. Performance of the CNN Model and Conventional Machine Learning Models

In the development set, the CNN model demonstrated strong discriminative performance based on out-of-fold predictions, with an AUC of 0.939 (95% CI: 0.913–0.962). Among the evaluated metrics, specificity and precision achieved high values exceeding 90%. They reached 0.917 (95% CI: 0.877–0.955) and 0.910 (95% CI: 0.867–0.950), respectively. In contrast, sensitivity demonstrated moderate results at 0.834 (95% CI 0.782–0.885). In the hold-out test set, the model maintained robust performance. The AUC was 0.974 (95% CI: 0.943–0.995), with an accuracy of 0.898 (95% CI: 0.837–0.949), sensitivity of 0.878 (95% CI: 0.781–0.959), and specificity of 0.918 (95% CI: 0.830–0.981) (Table 3).

For comparison, we evaluated the four conventional machine learning models using the same data split. In the development out-of-fold evaluation, LR achieved the highest AUC among the conventional models [0.928 (95% CI: 0.901–0.952)]. In the hold-out test set, LR again showed the highest AUC among the conventional models, with 0.911 (95% CI: 0.844–0.966). Other performance metrics of the machine learning model are presented in Table 3.

**Figure 4:**
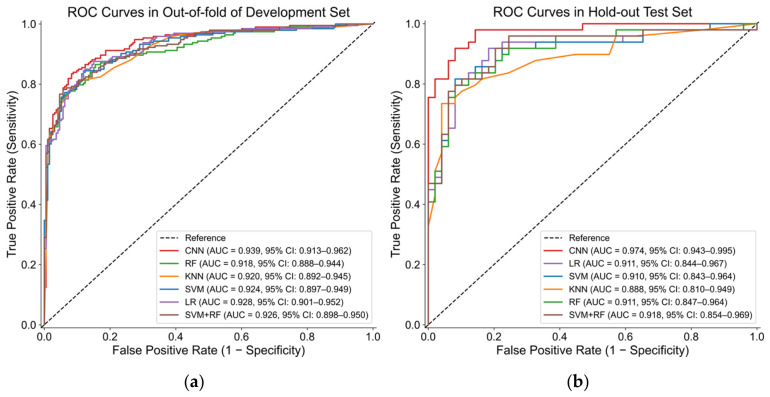
The ROC curves of different models in the development and hold-out test sets: (**a**) ROC curves in the out-of-fold development set; (**b**) ROC curves in the hold-out test set.

Given that the CNN model achieved the highest AUC in both the development and hold-out test sets, ROC curves were further generated to visually compare its discriminative ability with that of the conventional machine learning models (Figure 4a,b). In the hold-out test set, DeLong’s test indicated that the AUC of the CNN model was significantly higher than that of each machine learning model. After Holm–Bonferroni correction for multiple comparisons, all adjusted *p* values were <0.05 (Table S2).

**Figure 5:**
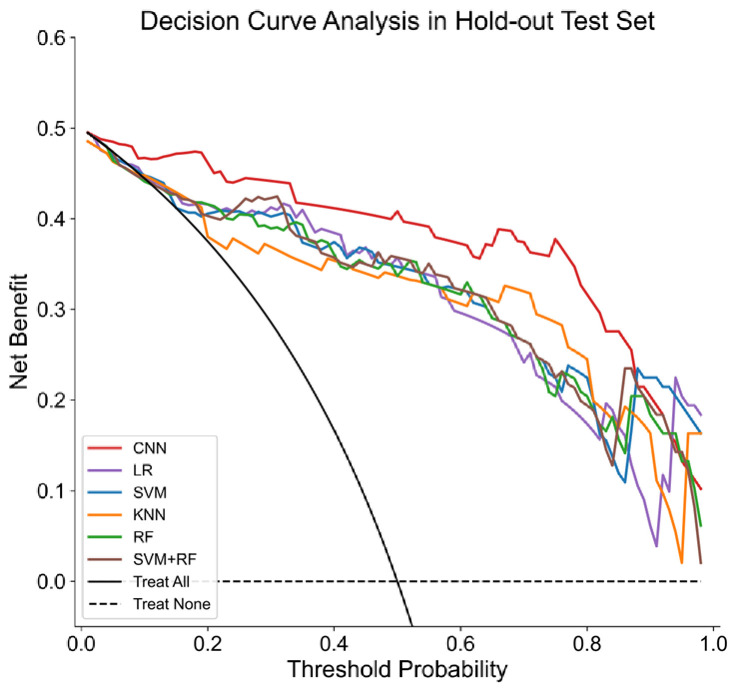
The DCA of different models in the hold-out test set.

Furthermore, DCA was conducted in the hold-out test set to assess the clinical usefulness of each model by quantifying the net benefit across different threshold probabilities. The results demonstrated that the CNN model achieved the highest net benefit across a wide range of threshold probabilities (<0.85). Because current clinical guidelines do not define a fixed probability threshold for RHC referral, we focused on an exploratory moderate risk threshold range of 0.2–0.4. This range was considered clinically plausible for symptomatic patients with suspected PH, in whom both missed PH and unnecessary RHC are relevant concerns. Across this threshold range, the CNN model provided the highest net benefit among the evaluated models (Figure 5).

**Figure 6:**
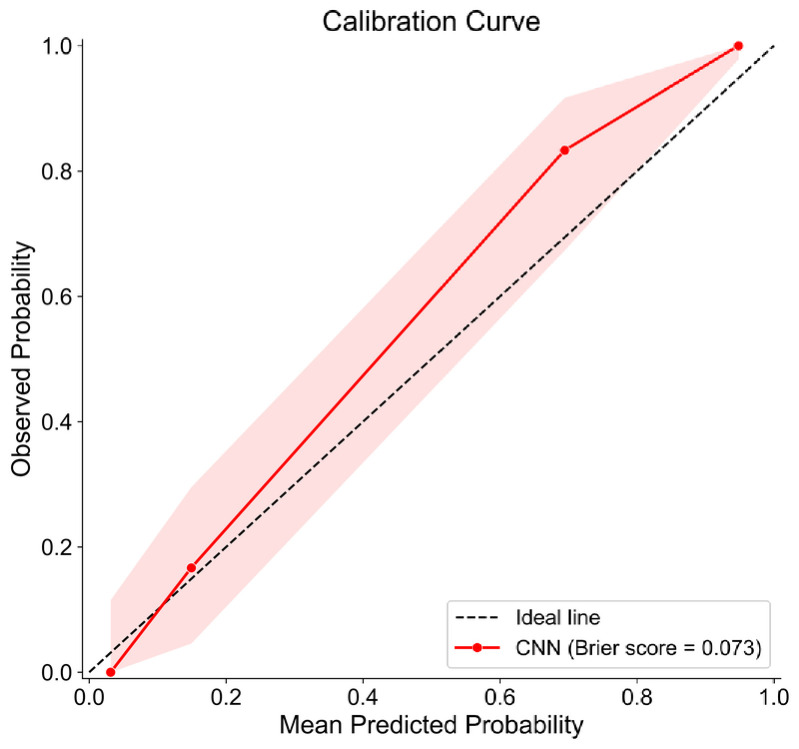
Calibration plot of the CNN model in the hold-out test set. The pink shaded area indicates the 95% confidence interval.

The calibration plot showed favorable agreement between the predicted probabilities and observed event probabilities in the hold-out test set, with a low Brier score of 0.073 (Figure 6). However, slight underestimation was observed in the intermediate-to-high probability range.

Overall, the CNN model achieved the best performance across both the development and hold-out test evaluations. These findings suggest that the CNN model may capture additional discriminative information from the 64-channel time-series signals and may offer improved generalization performance compared with conventional machine learning approaches.

### 3.3. Sensitivity and Ablation Analyses

In sensitivity analyses, two additional lightweight deep learning architectures, including a residual 1D-CNN and a multiscale 1D-CNN, were evaluated using the same data split and evaluation strategy. These models achieved comparable performance to the primary temporal CNN, supporting the robustness of direct raw MCG time-series modeling (Table S3).

Additionally, a temporal perturbation sensitivity analysis was performed to assess whether the performance of the CNN model depended on the temporal structure of the MCG waveform. As shown in Table 3, the trained final CNN model achieved an AUC of 0.974 on the hold-out test set. Temporal reversal reduced the AUC to 0.912, random time shuffling reduced the mean AUC to 0.960 ± 0.002, and the maximum sliding-window occlusion (QRS complex interval) reduced the AUC to 0.877 (Table S4). These results indicate that disruption of temporal morphology weakened model performance, supporting the contribution of cardiac cycle temporal information to CNN prediction.

In the hold-out test set, the temporal CNN model only based on MCG signals achieved an AUC of 0.981 (95% CI: 0.959–0.996), with sensitivity, specificity, precision, accuracy, and F1 score all reaching 0.918. The LR model built upon clinical covariates yielded only modest discriminative performance, with an AUC of 0.754 (95% CI: 0.648–0.849), suggesting that demographic variables alone could not explain the performance of the CNN models. The raw-signal principal component analysis (PCA) combined with machine learning models also achieved good performance, with hold-out AUCs of 0.963 (95% CI: 0.925–0.991) for SVM, 0.956 (95% CI: 0.913–0.988) for LR, and 0.960 (95% CI: 0.913–0.993) for RF (Table S5). These results indicate that the raw MCG time-series signal contains substantial discriminative information even when transformed into lower-dimensional PCA features.

### 3.4. Interpretation of the CNN Model

**Figure 7:**
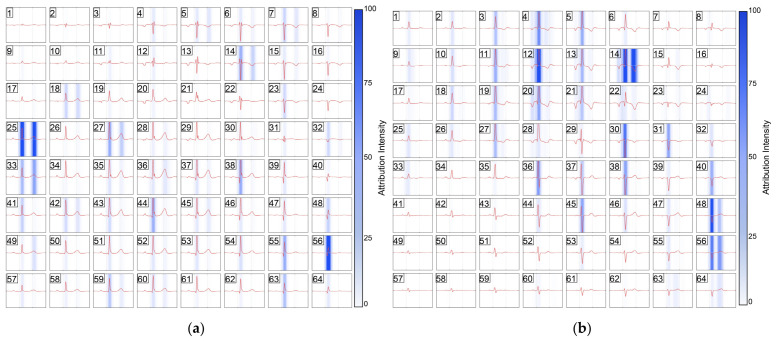
The attribution intensity maps of symptomatic controls and PH patients: (**a**) the attribution intensity maps of symptomatic controls; (**b**) the attribution intensity maps of PH patients. The red line represents a single cardiac cycle, and the numbers denote the corresponding sensor channels.

To interpret the proposed CNN model, we used the Integrated Gradients to trace the model’s predictions back to the original 64-channel time-series inputs (Figure 7). According to this method, we generated representative pure attribution maps for symptomatic controls and PH patients. In the controls, high-attribution regions were focal (Figure 7a). These regions were concentrated mainly in the left and lower left sections of the 64 channels time-series figures. In contrast, the high attribution intensity areas in PH patients were observed across multiple neighboring channels, which were distributed along the diagonal line spanning from channel 1 to channel 64, running from the top left to the bottom right (Figure 7b). A group-level statistical analysis of attribution patterns was further performed to determine whether the representative attribution patterns were consistent across the cohort. Attribution values were summarized as mean attribution intensity within P-wave, QRS-complex, and ST-T intervals. In both PH patients and controls, the QRS-complex interval showed the highest absolute mean attribution intensity. PH patients showed lower absolute mean attribution intensity than controls in the P-wave interval and QRS-complex interval, whereas no significant difference was observed in the ST-T interval (Table S6). These results support the presence of group-level attribution patterns in the hold-out cohort.

Regarding the position of attribution intensity across the cardiac cycle, symptomatic controls showed concentrations mainly in the QRS complex, and a small fraction in the T-wave interval. Conversely, areas of high attribution intensity in patients with PH could encompass the P wave, QRS complex, and the T wave across certain channels such as channel 14.

In summary, model predictions were associated with coordinated multichannel features across temporal regions corresponding to the P-wave, QRS complex, and ST-T intervals. These findings suggest that the model did not depend on an isolated amplitude peak or an abnormality in a single channel.

### 3.5. Spatial Redistribution of the QRS Complex

Because the attribution maps generated by Integrated Gradients suggested that QRS-related segments and their spatial distribution contributed substantially to model predictions, we further examined whether patients with PH demonstrated spatially distinct QRS morphology. The R/S amplitude ratio was calculated for each channel, and group-level median R/S ratio maps were generated for PH patients and controls.

**Figure 8:**
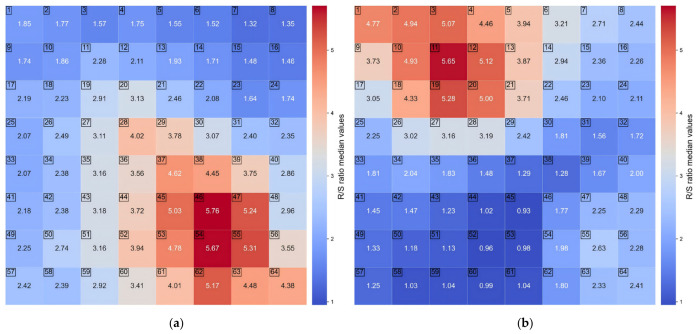
The R/S ratio heat maps of symptomatic controls and PH patients: (**a**) the R/S ratio heat maps of symptomatic controls; (**b**) the R/S ratio heat maps of PH patients. The displayed MCG map is a mirror-view projection relative to the subject’s body orientation; the upper-left region on the displayed map corresponds approximately to the subject’s upper-right thoracic region. The numbers denote the corresponding sensor channels.

The median R/S ratio maps showed a marked spatial redistribution between groups (Figure 8). In the symptomatic controls, the higher R/S values were predominantly located in the lower-middle to lower-right channels, with peak median values around channels 46 and 54 (Figure 8a). In contrast, PH patients showed a high-value cluster in the upper-left to upper-middle channels, with the highest median value around channel 11 (Figure 8b). Additionally, the highest median values exhibited no significant difference between the two groups. This pattern suggests that PH was associated with an altered spatial distribution of QRS morphology rather than a uniform increase in R/S amplitude ratio.

## 4. Discussion

In this study, we developed a CNN model to analyze 1D temporal signals from 64 channels MCG recordings in a single-center PH cohort enriched for PAH and CTEPH. Compared with conventional machine learning methods using 2D MCG-derived features, the proposed model showed improved detection performance.

Awareness and understanding of PH continue to grow, driving an increasing demand for accurate non-invasive assessment and detection technologies [2]. Current non-invasive detection methods primarily rely on indirect observation. They detect pathophysiological and electrophysiological cardiac alterations resulting from increased right ventricular load [6,17]. TTE and CMR serve as the only exceptions capable of estimating pulmonary artery pressure through valvular regurgitation or the reconstruction of pulmonary blood flow [18,19]. However, TTE is prone to substantial variability because it is influenced by operator experience and the degree of tricuspid regurgitation, whereas mature 4D flow CMR protocols are typically available only in research-oriented hospitals [20,21,22]. Therefore, developing an accurate and reliable non-invasive method for PH detection is urgently needed to enable earlier identification of PH, particularly in patients presenting with PH symptoms, such as chest tightness and exertional dyspnea.

With the development of quantum sensing technology, SERF-MCG has emerged as a potential modality for cardiac functional assessment. Owing to its high spatiotemporal sensitivity, MCG has been more widely explored for the early detection of ischemic heart disease [23,24]. Nevertheless, a limited number of studies have examined changes in MCG signals associated with right heart overload. In our previous works, feature parameters extracted from 2D MCG maps reconstructed using either 32-channel or 64-channel recordings, in combination with machine learning methods, showed good discriminatory performance for PH detection [10]. Moreover, MCG outperformed ECG and enabled the detection of PH even in patients with normal ECG findings [11].

In our previous work, SHAP analysis suggested that deviations in the bipolar orientation of the MF maps around the R-wave peak made the greatest contribution to model prediction, followed by changes in current intensity. However, an important limitation remains. The MCG feature parameters used in these studies were derived from PCD or MF maps reconstructed from 1D MCG signals. Although these maps offer greater visual interpretability, the interpolation and reconstruction steps may lead to varying degrees of information distortion and loss. Therefore, to avoid potential information loss during map reconstruction, 64-channel 1D MCG signals were analyzed directly in the present study. In this study, the 1 s MCG input was not an arbitrary segment directly selected from the 90 s recording, but a representative cardiac cycle template generated after preprocessing, R-peak localization, cardiac cycle segmentation, morphology-based beat selection, alignment, and averaging. This strategy was intended to reduce the influence of transient noise, atypical beats, respiratory variation, and beat-to-beat instability. At the same time, this method can preserve the dominant cardiac cycle temporal morphology of the MCG signal. Similar deep learning strategies have been explored in other unstable cardiovascular signals. Rezaee et al. proposed a graph convolutional network-based deep feature learning framework for cardiovascular disease recognition from heart sound signals, highlighting the value of extracting robust deep representations from local cardiovascular signal patterns [25]. Although heart sound signals and MCG signals differ substantially in physical origin and signal characteristics, both require strategies to handle local temporal variability before deep-learning-based classification.

Our results showed that the CNN model achieved the best overall performance in both the out-of-fold predictions from the development set and the hold-out test set. In the hold-out test set, the AUC of the CNN model exceeded that of the second-best model by 0.063 (0.974 vs. 0.911). In decision curve analysis, the CNN model also provided a higher net benefit across a broad range of threshold probabilities. Taken together, these findings suggest that direct modeling of raw MCG time-series signals may preserve discriminative information that is not fully captured by reconstructed map-derived features. A similar finding has been reported in ECG, where direct analysis of raw ECG signals using deep learning improved PH detection and achieved higher performance than conventional machine learning methods [13,14,26,27].

The supplementary ablation analyses could clarify the role of clinical covariates and MCG time-series signals in model performance. Age, sex, and BMI differed significantly between PH patients and controls, indicating that these variables may contain cohort information. Therefore, age, sex, and BMI were included as clinical covariates in the primary temporal CNN and ML models to incorporate basic demographic information. However, the LR model based on the three variables achieved only moderate performance. In contrast, the temporal CNN only based on MCG achieved a hold-out AUC of 0.981, comparable to the primary CNN model. These findings suggest that the main discriminative information was derived from the raw MCG time-series signal.

In the sensitivity and ablation analyses, the raw-signal PCA + ML models achieved good performance. Moreover, temporal perturbation and sliding occlusion analyses further indicated that the CNN relied on cardiac cycle temporal morphology rather than only demographic information or static amplitude differences. Additional residual 1D-CNN and multiscale 1D-CNN analyses showed comparable performance. These results further support the diagnostic information contained in the original 64-channel MCG time series.

Machine learning models, particularly more complex deep learning models, remain limited by insufficient interpretability in clinical applications. Therefore, improving model interpretability is important for enhancing clinical trust and clinical translation [28,29,30]. In this study, we applied Integrated Gradients to generate attribution maps, allowing us to examine which temporal segments and input channels were most influential in the CNN model predictions. Attribution maps from representative subjects showed that PH prediction by the model was driven by features distributed across multiple channels, and high-attribution regions appeared in a clustered pattern in the PH patient map. Moreover, high-contribution regions within the P-wave interval were not observed in the attribution maps of controls. These attribution regions overlapped with the P-wave interval, suggesting that the model used signal components occurring during this phase of the cardiac cycle. In addition, although the ST-T segment was highlighted with varying intensity in both PH and control maps, this may be attributable to the presence of coronary artery disease in a proportion of the symptomatic controls. We found that in PH patients, the attribution maps showed that the high attribution intensity regions were concentrated mainly in the QRS complex. This pattern suggests that the model was highly sensitive to signal morphology during the QRS-complex interval. However, because Integrated Gradients merely identifies temporal samples and channels contributing to model predictions, Integrated Gradients alone cannot directly localize the underlying cardiac electrical source. Consequently, the interpretation of the CNN model should be regarded as hypothesis-generating. Therefore, the attribution regions corresponding to the P-wave, QRS-complex, and T-wave intervals should be interpreted as model-sensitive cardiac cycle segments rather than definitive evidence of atrial or ventricular electrophysiological abnormalities. The R/S ratio heat maps analysis provided complementary signal-level evidence of altered QRS spatial morphology, but it also represents a surface projection of magnetic field components rather than anatomical source localization.

In this study, high R/S ratio regions in symptomatic controls were found mainly in the lower-left area of the sensor panel, which was consistent with the left ventricular and apical regions described in previous MCG studies [31]. By contrast, in patients with PH, the high R/S ratio regions were shifted to the upper-left area of the panel, suggesting that right heart loading related to PH may alter the spatial distribution of the cardiac magnetic field during the QRS complex. The SERF-OPM sensors used in this study were single-axis sensors and recorded the *z*-axis component of the cardiac magnetic field (perpendicular to the chest wall). Therefore, according to the basis of the magnetic field distribution pattern, we speculate that the R/S ratio heat map should be interpreted as a surface projection of ventricular depolarization magnetic field components, rather than as direct anatomical localization of the electrical source. Given that source localization in MCG remains at an early stage, this represents an important direction for our future research.

From the clinical perspective, the proposed CNN model should be interpreted as a complementary, non-invasive triage tool rather than a replacement for TTE or RHC. RHC remains the diagnostic gold standard for PH, and TTE remains the primary non-invasive modality for estimating the probability of PH. The potential role of the model combined with MCG is to provide additional electrophysiological information in symptomatic patients with suspected PH and to help identify patients who may benefit from further RHC evaluation. Therefore, the model is best positioned as a supplementary screening adjunct before invasive confirmation, rather than as a stand-alone diagnostic test.

There are several limitations in this study. First, this was a single-center exploratory study. Although rigorous cross-validation was performed and a hold-out test set was used for model evaluation, external validation was not available, which may limit the generalizability of the model. Therefore, the generalizability of the model requires further confirmation. Second, the study cohort may have introduced spectrum bias. In real clinical settings, Group 2 and Group 3 account for a major fraction of all underlying causes for PH, whereas the PH cohort in this study was composed mainly of patients with PAH and CTEPH. This may limit the applicability of the model to broader PH populations. Additionally, this may potentially prevent the CNN model from fully capturing the comprehensive spectrum of PH-specific MCG features. Therefore, multi-center studies with larger cohorts and more diverse PH etiologies are needed to evaluate the stability and clinical applicability of the model. Furthermore, the majority of controls exhibited symptoms similar to PH but did not undergo RHC for definitive exclusion of PH, which may introduce misclassification bias into the control group. At the same time, the demographic imbalance between groups remains an important limitation. Future studies should validate the model in independent prospective external cohorts with broader clinical spectra to further assess the independence and generalizability of MCG-based prediction. Finally, a proportion of PH patients had received PAH-targeted therapy before enrolment, which may have influenced MCG results and model predictions. Future works should prioritize newly diagnosed, treatment-naive patients with PH to minimize the potential effects of treatment.

## 5. Conclusions

In recent years, SERF-OPM-based MCG has shown promise as an important non-invasive tool for cardiovascular disease detection due to its high spatiotemporal sensitivity. In this single-center exploratory study, a CNN model for PH detection was developed using 1D signals from 64-channel MCG recordings. The model showed promising internal performance for PH detection in a cohort enriched for PAH and CTEPH, achieving an AUC of 0.974 in the internal hold-out test set and outperforming conventional machine learning methods. However, this study remains exploratory; the performance of the model and its applicability to the broader PH population require further validation in larger, multi-center PH cohorts.

## Figures and Tables

**Table 1:** The characteristic of PH patients.

Characteristics	PH Patients
Female, n (%)	189 (78.1%)
Age, years	43.04 ± 15.06
BMI, kg/m^2^	22.96 ± 3.53
WHO-FC, n (%)	
I/II	173 (71.5%)
III/IV	69 (28.5%)
6MWD, m	443.62 ± 99.28
NT-proBNP, pg/mL	229.50 (71.35, 780.50)
PH classification	
IPAH	74 (30.6%)
CHD-PAH	68 (28.1%)
CTD-PAH	43 (17.8%)
PoPAH	4 (1.7%)
CTEPH	44 (18.2%)
LHD-PH	6 (2.5%)
LD-PH	3 (1.2%)
RHC	
mRAP, mmHg	4.00 (3.00, 6.00)
mRVP, mmHg	25.52 ± 9.31
mPAP, mmHg	45.67 ± 15.52
PAWP, mmHg	6.00 (5.00, 9.00)
CO, mL/min	4.71 ± 1.40
CI, mL/min/m^2^	2.94 ± 0.87
PVR, Wood units	7.25 (4.55, 11.33)
SvO_2_, %	67.24 ± 11.51

6MWD, 6 min walk distance; BMI, body mass index; CHD-PAH, congenital heart disease-associated pulmonary arterial hypertension; CI, cardiac index; CO, cardiac output; CTD-PAH, connective tissue disease-associated pulmonary arterial hypertension; CTEPH, chronic thromboembolic pulmonary hypertension; IPAH, idiopathic pulmonary arterial hypertension; LD-PH, PH associated with lung disease; LHD-PH, PH associated with left heart disease; mPAP, mean pulmonary arterial pressure; mRAP, mean right atrial pressure; mRVP, mean right ventricular pressure; NT-proBNP, N-terminal pro-B-type natriuretic peptide; PAWP, pulmonary arterial wedge pressure; PH, pulmonary hypertension; PoPAH, portal hypertension-associated pulmonary arterial hypertension; PVR, pulmonary vascular resistance; RHC, right heart catheterization; SvO_2_, mixed venous oxygen saturation; WHO-FC, World Health Organization functional class.

**Table 2:** The TTE parameters between PH patients and controls.

TTE Parameters	PH Patients	Controls	*p* Values
LAd, mm	30.33 ± 6.50	35.35 ± 4.92	<0.001
LVd, mm	37.56 ± 7.90	46.72 ± 7.20	<0.001
RAa, mm^2^	19.22 ± 8.02	14.27 ± 3.36	<0.001
RVd, mm	31.85 ± 8.57	23.47 ± 2.94	<0.001
mPA, mm	32.63 ± 7.29	22.72 ± 2.75	<0.001
LVEI	1.34 (1.02, 1.62)	\	\
TAPSE, mm	18.47 ± 4.21	\	\
RVOT-AT, ms	71.51 ± 18.65	\	\
TRV, m/s	4.04 ± 0.69	\	\
sPAP, mmHg	65.50 (55.00, 86.00)	23.88 ± 4.66	<0.001

LAd, left atrial diameter; LVd, left ventricular diameter; LVEI, left ventricular eccentricity index; mPA, main pulmonary artery diameter; RAa, right atrial area; RVd, right ventricular diameter; RVOT-AT, right ventricular outflow tract acceleration time; sPAP, systolic pulmonary arterial pressure; TAPSE, tricuspid annular plane systolic excursion; TRV, tricuspid regurgitation velocity; TTE, transthoracic echocardiography.

**Table 3:** The different models’ performance in the development and hold-out test sets.

Models	Sen	Spe	Precision	Acc	F1 Score	AUC
Out-of-fold in development set						
CNN	0.834 (0.782–0.885)	0.917 (0.877–0.955)	0.910 (0.867–0.950)	0.876 (0.842–0.909)	0.870 (0.832–0.906)	0.939 (0.913–0.962)
KNN	0.782 (0.724–0.840)	0.917 (0.875–0.954)	0.904 (0.858–0.948)	0.850 (0.813–0.883)	0.839 (0.795–0.877)	0.920 (0.892–0.945)
LR	0.845 (0.791–0.891)	0.880 (0.832–0.924)	0.876 (0.827–0.922)	0.863 (0.826–0.894)	0.860 (0.820–0.895)	0.928 (0.901–0.952)
RF	0.865 (0.813–0.912)	0.845 (0.793–0.892)	0.848 (0.795–0.895)	0.855 (0.819–0.889)	0.856 (0.815–0.891)	0.918 (0.888–0.944)
SVM	0.834 (0.780–0.883)	0.886 (0.839–0.929)	0.880 (0.832–0.924)	0.860 (0.824–0.894)	0.856 (0.816–0.891)	0.924 (0.897–0.949)
SVM + RF	0.782 (0.725–0.837)	0.943 (0.908–0.974)	0.932 (0.893–0.968)	0.863 (0.826–0.894)	0.851 (0.810–0.887)	0.926 (0.898–0.950)
Hold-out test set						
CNN	0.878 (0.780–0.959)	0.918 (0.830–0.981)	0.915 (0.830–0.981)	0.898 (0.837–0.949)	0.896 (0.826–0.952)	0.974 (0.943–0.995)
KNN	0.755 (0.634–0.867)	0.918 (0.833–0.981)	0.902 (0.805–0.977)	0.837 (0.755–0.908)	0.822 (0.727–0.899)	0.888 (0.815–0.952)
LR	0.837 (0.725–0.933)	0.878 (0.778–0.961)	0.872 (0.773–0.959)	0.857 (0.786–0.918)	0.854 (0.769–0.922)	0.911 (0.844–0.966)
RF	0.837 (0.729–0.933)	0.796 (0.674–0.906)	0.804 (0.689–0.907)	0.816 (0.735–0.888)	0.820 (0.727–0.894)	0.911 (0.848–0.964)
SVM	0.776 (0.646–0.889)	0.918 (0.833–0.981)	0.902 (0.805–0.977)	0.847 (0.776–0.918)	0.835 (0.741–0.911)	0.910 (0.846–0.967)
SVM + RF	0.755 (0.627–0.875)	0.939 (0.864–1.000)	0.925 (0.833–1.000)	0.847 (0.776–0.918)	0.831 (0.738–0.911)	0.918 (0.854–0.969)

Acc, accuracy; AUC, area under curve; CNN, convolutional neural network; KNN, k-nearest neighbor; LR, logistic regression; RF, random forest; Sen, sensitivity; Spe, specificity; SVM, support vector machine.

## Data Availability

The raw MCG data of the patients in this study cannot be made publicly available because of privacy. Due to ethical restrictions, de-identified clinical data and MCG features data in this study are available on request from the corresponding author.

## Supplementary Materials

The following supporting information can be downloaded at: https://www.mdpi.com/article/10.3390/bioengineering13070736/s1, Table S1. The baseline clinical covariates between two groups; Table S2. The DeLong test of the CNN model vs. other machine learning models; Table S3. The results of different deep learning models; Table S4. The results of temporal perturbation sensitivity analysis; Table S5. The results of ablation tests; Table S6. The group-level statistical analysis of attribution patterns.
