# Supplementary material for: Temporal Convolutional Neural Network Analysis of Magnetocardiography Signals for Detection of Pulmonary Hypertension
Source: Bioengineering (Basel). 2026 Jun 25;13(7):736. doi: 10.3390/bioengineering13070736 (PMC13405781; doi:10.3390/bioengineering13070736)
Supplement: Supplementary file 1 [file bioengineering-13-00736-s001.zip › bioengineering-4340626-supplementary.pdf]

Supplementary Materials

Supplementary Methods

CNN modeling

A binary classification model with two input branches was developed, consisting of an MCG time-series convolutional branch and a clinical covariate branch including sex, age, and BMI. Each MCG input sample was represented as a 1 × 1000 × 64 tensor, corresponding to one cardiac-cycle segment, 1000 temporal samples, and 64 MCG channels. The MCG branch consisted of three temporal convolutional blocks. The first block included a convolutional layer with 32 filters and a kernel size of 1 × 9, followed by batch normalization, Rectified Linear Unit (ReLU) activation, and max pooling with a pool size of 1 × 2. The second block included a convolutional layer with 64 filters and a kernel size of 1 × 7, followed by batch normalization, ReLU activation, and max pooling with a pool size of 1 × 2. The third block included a convolutional layer with 128 filters and a kernel size of 1 × 5, followed by batch normalization and ReLU activation. A dropout layer with a dropout rate of 0.20 was then applied, followed by global average pooling to obtain the MCG feature vector. The MCG feature vector was concatenated with the standardized clinical covariates and passed to a single-neuron fully connected layer to obtain the binary classification logit. A sigmoid function was subsequently applied to generate the predicted probability.

The model was trained using the binary cross-entropy loss function and the Adam optimizer. The maximum number of training epochs was 60, with a mini-batch size of 16 and an initial learning rate of 1 × 10-3. The learning rate was multiplied by 0.30 every 15 epochs. L2 weight decay was set to 1 × 10-4, and gradient clipping was applied with an L2-norm threshold of 5.0. During inner cross-validation, early stopping was applied based primarily on validation AUC, with validation loss used as a secondary criterion when validation AUC was unchanged. Training was stopped if validation AUC did not improve by at least 1 × 10-4 for 10 consecutive epochs after a minimum of 15 epochs. After inner five-fold cross-validation, the final number of

training epochs was determined as the median of the best epochs across the five folds, with a minimum of five epochs. The final model was then retrained on the entire development set using this fixed number of epochs and evaluated once on the hold-out test set.

The model interpretation was conducted on the hold-out test set. Because the proposed model included both MCG time-series inputs and clinical covariates, the clinical covariates, including sex, age, and BMI, were kept fixed when calculating attributions for the MCG time-series branch. Integrated Gradients was computed in the standardized model-input space using 32 interpolation steps. A zero baseline was used, corresponding to the development-set mean level after z-score normalization.

The modeling methods of additional deep learning algorithm

Residual 1D-CNN

The input MCG tensor first passed through a temporal convolutional stem consisting of a 1 × 7 convolutional layer with 32 filters, followed by batch normalization and ReLU activation. The first residual block contained two 1 × 5 convolutional layers with 32 filters. The input to this block was connected directly to the output of the second convolution through an identity residual connection, followed by ReLU activation. The filters in the second residual block was 64 and the block reduced the temporal resolution using a stride of 2 in the first 1 × 5 convolution. Due to the feature dimension changed, the shortcut branch used a 1 × 1 convolution with stride 2, followed by batch normalization, to match the dimensions of the main branch before residual addition. The third residual block further increased the number of filters to 128 and again reduced the temporal resolution using a stride of 2. This block used two 1 × 3 convolutional layers in the main branch and a 1 × 1 strided convolution in the shortcut branch.

After the final residual block, ReLU activation, dropout with a rate of 0.20, and global average pooling were applied to obtain the MCG feature vector.

Multiscale 1D-CNN

The initial multiscale block contained four parallel temporal convolutional

branches with kernel sizes of 1 × 5, 1 × 11, 1 × 21, and 1 × 1. Each branch used 24 filters. The 1 × 5 branch was intended to capture short local waveform changes, the 1 × 11 and 1 × 21 branches were used to capture wider temporal morphology, and the 1 × 1 branch provided channel-wise feature mixing. The outputs of the four branches were concatenated along the channel dimension, followed by batch normalization and ReLU activation. The second multiscale block also contained four parallel branches, with kernel sizes of 1 × 3, 1 × 7, 1 × 15, and 1 × 1. Each branch used 32 filters. The outputs were concatenated, followed by batch normalization, ReLU activation, dropout with a rate of 0.20, and global average pooling. The resulting MCG feature vector was concatenated with the same standardized clinical covariates and passed to a single-neuron fully connected layer to obtain the binary classification logit. The final predicted probability was generated using a sigmoid function.

The methods of ablation analyses

First, an MCG-only temporal CNN was trained using only the raw 64-channel MCG time-series input, without age, sex, or BMI. Second, a clinical covariates-only logistic regression model was constructed using only age, sex, and BMI. Third, to construct input-comparable shallow-learning baselines, we developed raw-signal principal component analysis (PCA) combined with conventional machine learning models using the same 64 × 1000 representative MCG time-series template as the CNN input. Within each inner training fold, MCG signals were standardized channel-wise using only the training samples. Each standardized 64 × 1000 template was then flattened into a 64,000-dimensional vector. PCA was fitted only on the training fold to reduce dimensionality, and the fitted PCA transformation was applied to the validation fold or hold-out test set to avoid information leakage. The number of PCA components was selected to explain approximately 95% of the cumulative variance, with a maximum of 50 components. The PCA scores were concatenated with standardized age, sex, and BMI, and used to train LR, SVM, and RF classifiers. The same five-fold out-of-fold prediction procedure, Youden-index threshold selection, and hold-out test evaluation strategy were used as in the primary CNN analysis. For the final hold-out test evaluation, the normalization, PCA transformation,

covariate standardization, and classifier were refitted using the entire development set and then applied once to the hold-out test set.

Supplementary Table

Table S1. The baseline clinical covariates between two groups

Clinical covariates	PH patients	Controls	*p* value
Female, n (%)	189 (78.1%)	53 (26.8%)	< 0.001
Age, years	43.04 ± 15.06	51.65 ± 12.32	< 0.001
BMI, kg/m^2	22.96 ± 3.53	25.31 ± 3.83	< 0.001

Table S2. The DeLong test of the CNN model vs. other machine learning models.

Comparison	ΔAUC	z value	*p* value	adjusted *p* value
CNN vs. RF	0.063	2.50	0.012	0.037
CNN vs. KNN	0.086	2.86	0.004	0.017
CNN vs. SVM	0.063	2.44	0.015	0.037
CNN vs. LR	0.062	2.36	0.018	0.037
CNN vs. SVM+RF	0.056	2.32	0.021	0.049

Table S3. The results of different deep learning models

Models	Sen	Spe	Precision	Acc	F1 score	AUC
Out-of-fold in development set						
Primary CNN	0.834 (0.782-0.885)	0.917 (0.877-0.955)	0.910 (0.867-0.950)	0.876 (0.842-0.909)	0.870 (0.832-0.906)	0.939 (0.913-0.962)
Residual 1D-CNN	0.839 (0.785-0.887)	0.917 (0.877-0.953)	0.910 (0.869-0.949)	0.878 (0.845-0.909)	0.873 (0.836-0.907)	0.937 (0.911-0.960)
Multiscale 1D-CNN	0.902 (0.856-0.939)	0.928 (0.890-0.963)	0.926 (0.886-0.961)	0.915 (0.883-0.940)	0.913 (0.881-0.940)	0.941 (0.911-0.964)
Hold-out test set						
Primary CNN	0.878 (0.780-0.959)	0.918 (0.830-0.981)	0.915 (0.830-0.981)	0.898 (0.837-0.949)	0.896 (0.826-0.952)	0.974 (0.943-0.995)
Residual 1D-CNN	0.878 (0.780-0.960)	0.959 (0.894-1.000)	0.956 (0.889-1.000)	0.918 (0.851-0.969)	0.915 (0.848-0.968)	0.974 (0.943-0.995)
Multiscale 1D-CNN	0.939 (0.861-1.000)	0.898 (0.808-0.978)	0.902 (0.811-0.979)	0.918 (0.857-0.969)	0.920 (0.857-0.969)	0.969 (0.934-0.992)

Table S4. The results of temporal perturbation sensitivity analysis

Temporal analysis	AUC	ΔAUC
Original input	0.974	/
Time reversal	0.912	0.061
Random time shuffling	0.960 ± 0.002	0.014
Maximum sliding-window occlusion	0.877	0.097

Table S5. The results of ablation tests

Models	Sen	Spe	Precision	Acc	F1 score	AUC
Out-of-fold in development set						
Temporal CNN with clinical covariates	0.834 (0.782-0.885)	0.917 (0.877-0.955)	0.910 (0.867-0.950)	0.876 (0.842-0.909)	0.870 (0.832-0.906)	0.939 (0.913-0.962)
MCG-only temporal CNN	0.860 (0.809-0.910)	0.886 (0.842-0.929)	0.883 (0.837-0.927)	0.873 (0.839-0.904)	0.871 (0.834-0.906)	0.930 (0.903-0.955)
Clinical covariates model	0.746 (0.687-0.806)	0.725 (0.663-0.791)	0.731 (0.671-0.793)	0.736 (0.694-0.780)	0.739 (0.690-0.787)	0.783 (0.737-0.831)
Raw-signal PCA + SVM	0.845 (0.791-0.896)	0.938 (0.902-0.969)	0.931 (0.891-0.966)	0.891 (0.858-0.922)	0.886 (0.849-0.919)	0.911 (0.876-0.942)
Raw-signal PCA + LR	0.823 (0.770-0.879)	0.959 (0.930-0.985)	0.952 (0.920-0.982)	0.891 (0.860-0.922)	0.883 (0.846-0.918)	0.910 (0.876-0.942)
Raw-signal PCA + RF	0.824 (0.766-0.875)	0.933 (0.897-0.967)	0.924 (0.884-0.962)	0.878 (0.842-0.909)	0.871 (0.832-0.905)	0.938 (0.913-0.960)
Hold-out test set						
Temporal CNN with clinical covariates	0.878 (0.780-0.959)	0.918 (0.830-0.981)	0.915 (0.830-0.981)	0.898 (0.837-0.949)	0.896 (0.826-0.952)	0.974 (0.943-0.995)
MCG-only temporal CNN	0.918 (0.840-0.981)	0.918 (0.830-0.981)	0.918 (0.837-0.982)	0.918 (0.867-0.969)	0.918 (0.857-0.969)	0.981 (0.959-0.996)
Clinical covariates model	0.694 (0.566-0.821)	0.714 (0.582-0.830)	0.708 (0.583-0.830)	0.704 (0.612-0.796)	0.701 (0.590-0.796)	0.754 (0.648-0.849)
Raw-signal PCA + SVM	0.918 (0.833-0.982)	0.878 (0.781-0.961)	0.882 (0.786-0.962)	0.898 (0.837-0.949)	0.900 (0.830-0.956)	0.963 (0.925-0.991)
Raw-signal PCA + LR	0.857 (0.750-0.946)	0.918 (0.833-0.981)	0.913 (0.822-0.980)	0.888 (0.816-0.949)	0.884 (0.808-0.946)	0.956 (0.913-0.988)
Raw-signal PCA + RF	0.918 (0.830-0.980)	0.918 (0.836-0.981)	0.918 (0.833-0.981)	0.918 (0.857-0.969)	0.918 (0.852-0.968)	0.960 (0.913-0.993)

Table S6. The group-level statistical analysis of attribution patterns.

Attribution type	Interval	Controls, median mean attribution intensity	PH patients, median mean attribution intensity	*p* value*
Absolute	P-wave	0.000578	0.000462	**0.019**
Absolute	QRS	0.002223	0.001773	**0.012**
Absolute	ST-T	0.000787	0.000791	0.629
Positive	P-wave	0.000289	0.000245	**0.019**
Positive	QRS	0.001100	0.000834	**0.002**
Positive	ST-T	0.000401	0.000398	0.629
Negative	P-wave	0.000284	0.000219	**0.019**
Negative	QRS	0.001053	0.000920	**0.027**
Negative	ST-T	0.000376	0.000385	0.586

*， *p* values were adjusted using the Benjamini-Hochberg method.
